# Biomarker enhanced risk prediction for development of AKI after cardiac surgery

**DOI:** 10.1186/s12882-018-0902-9

**Published:** 2018-05-02

**Authors:** Michael L. Merchant, Michael E. Brier, Mark S. Slaughter, Jon B. Klein, Kenneth R. McLeish

**Affiliations:** 1 Division of Nephrology & Hypertension, Department of Medicine, University of Louisville School of Medicine, Donald Baxter Research Building, Rm 204C, 570 S. Preston Street, Louisville, KY 40202 USA; 2 Cardiovascular and Thoracic Surgery, University of Louisville School of Medicine, Louisville, USA; 3 Robley Rex VAMC, University of Louisville School of Medicine, Louisville, KY USA

**Keywords:** AKI, Cardiac surgery, Urine, Biomarker, Prognostic

## Abstract

**Background:**

Acute kidney injury (AKI) is a common post-cardiac surgery complication and influences patient morbidity and prognosis. This study was designed to identify preoperative candidate urine biomarkers in patients undergoing cardiac surgery.

**Methods:**

A prospective cohort study of adults undergoing cardiac surgery at increased risk for AKI at a single hospital between July 2010 and September 2012 was performed. The primary outcome was the development of AKI, defined as an absolute serum creatinine (SCr) level increase ≥ 0.5 mg/dL or a ≥ 50% relative increase within 72 h of surgery. A secondary outcome was development of AKI defined by Kidney Disease Improving Global Outcomes (KDIGO). Urine collected by voiding within 4 h prior to surgery was used for proteomic analysis and confirmatory enzyme linked immunosorbent assays (ELISAs) studies. Biomarkers were tested for AKI-prediction using Cox and Snell R^2^, area under the receiver operating curve (AUROC), and percent of corrected classifications. To evaluate the added effect of each candidate biomarker on AKI discrimination, receiver operator characteristic (ROC) curves, integrated discrimination improvement (IDI), and net reclassification improvement (NRI) were calculated.

**Results:**

Forty-seven of 755 patients met screening criteria including 15 with AKI. Proteomic analysis identified 29 proteins with a significant ≥2-fold change. Confirmatory ELISA measurements of five candidate markers showed urinary complement factor B (CFB) and histidine rich glycoprotein (HRG) concentrations were significantly increased in patients with AKI. By multivariate analysis, NRI, and IDI the addition of CFB and HRG to the standard clinical assessment significantly improved risk prediction for the primary outcome. Only HRG was a significant predictor in the 21 patients with AKI defined by KDIGO criteria.

**Conclusions:**

Pre-operative urine measurement of CFB or HRG significantly enhanced the current post-surgery AKI risk stratification for more restrictive definition of AKI. HRG, but not CFB or clinical risk stratification, predicted AKI defined by KDIGO. The ability of these biomarkers to predict risk for dialysis-requiring AKI or death could not be reliably assessed in our study due to a small number of patients with either outcome.

**Electronic supplementary material:**

The online version of this article (10.1186/s12882-018-0902-9) contains supplementary material, which is available to authorized users.

## Background

Acute kidney injury (AKI) is a common and serious complication after cardiac surgery. Using standardized definitions of AKI based primarily on an increased serum creatinine (SCr), 10% to 40% of patients undergoing cardiac surgery develop AKI [1–7]. AKI after cardiac surgery is associated with increased short-term and long-term mortality, increases in length of ICU and hospital stay, ventilator days, cost of hospitalization, and risk of developing chronic kidney disease (CKD) and end-stage renal disease (ESRD) [8–11]. Staging AKI according to RIFLE (Risk, Injury, Failure, Loss of kidney function, and End-stage kidney disease), AKIN (Acute Kidney Injury Network), and/or KDIGO (Kidney Disease Improving Global Outcomes) criteria showed that even mild forms of AKI not requiring dialysis are associated with increased morbidity, mortality, and risk of CKD [8, 12–15]. The diagnosis of AKI primarily depends on an increase in SCr concentration, which typically does not occur until 24 h to 72 h after injury [16]. The delay in diagnosis until injury contributes to the failure in human trials to reproduce successful interventions of experimental animal models [17, 18]. Without effective treatment of AKI, clinical management focuses on prevention and risk management.

Due to the importance of post-cardiac surgery AKI and influence on patient morbidity and prognosis, it is critical that patients and providers have a realistic pre-surgical understanding of AKI risk. Preoperative risk stratification for AKI after cardiac surgery is necessary for clinical decision making, for pre- and intra-operative treatment to minimize the risk of AKI, and to identify high-risk patients for clinical trials. A model developed at the Cleveland Clinic, using a combination of laboratory (including SCr) and clinical findings [19], was reported to best predict cardiac surgery-related AKI [3, 20]. That model, however, was developed to predict the risk of AKI requiring dialysis, and its ability to predict AKI of less severity is more limited [21]. Kiers et al. [3] reported an area under receiver operating curve (AUROC) of 0.75 for AKI-Risk and 0.81 for AKI-Injury, compared to an AUROC of 0.93 for AKI requiring dialysis.

A number of urine and blood biomarkers, including neutrophil gelatinase-associated lipocalin (NGAL), interleukin-18, cystatin C, and kidney injury molecule-1 (KIM-1), increase before SCr, improving the early diagnosis of AKI [4, 22–26]. Addition of pre-operative cystatin C in place of SCr to the clinical AKI risk assessment was reported to modestly improve risk stratification [7]. Identification of new biomarkers that replace or enhance current clinical risk stratification is needed to allow clinicians to apply appropriate preventive measures and to design clinical trials to identify effective therapies. The purpose of the present study was to identify preoperative candidate urine biomarkers in patients undergoing cardiac surgery that, alone or in combination with the current clinical scoring tool, would improve prediction of AKI. The data indicate that addition of preoperative urine HRG or complement factor B to the clinical scoring tools may improve the accuracy of prediction of AKI after open-heart surgery [19, 27].

## Methods

### Study population

The primary objective of this study was to identify pre-surgical biomarkers for AKI. We performed a prospective cohort study of adults undergoing cardiac surgery (coronary artery bypass grafting [CABG], surgery for valve disease, and both) at the Jewish Hospital of Louisville between July 2010 and September 2012 who were at risk for AKI. Risk for AKI was defined as a risk score of 5 or greater, based on the clinical algorithm published by Thakar et al. [19]. Exclusion criteria included age less than 18 years, stage 5 CKD or end stage renal disease, oliguria prior to surgery, fluctuations in SCr greater than 25% or 0.5 mg/dl prior to surgery, pregnancy, or inability to provide informed consent. Clinical data and measurement of baseline SCr were obtained on each subject within 2 days prior to surgery. Post-surgical clinical data and blood draws used for SCr measurement were obtained at 24 h, 48 h, and 72 h after surgery. Urine output after surgery was recorded at 2 h, 6 h and 24 h. Clinical data included demographics (age, sex, and race), comorbid conditions (hypertension, diabetes mellitus, heart failure, prior myocardial infarction, chronic obstructive pulmonary disease, peripheral vascular disease, stroke), surgery characteristics (elective or urgent; bypass, valvular surgery, or both; prior cardiac operation), and medications. Patients requiring emergent surgery were excluded from this study. SCr was measured in the hospital’s clinical laboratory, using a modified Jaffé assay. This study was approved by the Human Studies Committees at the Robley Rex VAMC and the University of Louisville, School of Medicine and adheres to the Declaration of Helsinki. A total of 67 patients of the 755 patients screened met eligibility requirements and provided written informed consent. Of those 67 patients, 12 failed to meet eligibility during pre-operative laboratory evaluation, and surgery was canceled for 4 patients. The remaining 51 patients form the study population.

### Outcome definitions

The primary outcome was the development of AKI, defined as an absolute SCr level increase ≥0.5 mg/dL or a ≥ 50% relative increase within 72 h of surgery when compared to the baseline SCr determined in all subjects prior to surgery. A definition of AKI more restrictive than that used by AKIN or KDIGO was employed (AKI_R_), as changes in fluid balance after cardiac surgery may lead to over diagnosis of AKI [28]. AKI as defined by KDIGO, an increase in SCr of 0.3 mg/dl within 48 h of surgery or a reduction in urine output to less than 0.5 ml/kg/h for 6 h, was determined as a secondary outcome (AKI_KDIGO_). All baseline SCr values were measured within 2 days prior to surgery. A total of 15 patients satisfied criteria for AKI_R_.

### Sample collection

Urine (50 ml) was collected by voiding within 4 h prior to surgery. Fresh urine samples were added to 50 ml tubes containing a protease inhibitor (Roche) and centrifuged at 1200 g for 15 min at 4 °C to remove cellular debris. Supernatants were aliquoted into 15 ml vials and stored at − 80 °C until use.

### AKI biomarker measurements

Personnel performing the biomarker measurements were blinded to each patient’s clinical information. All biomarkers were measured from frozen aliquots that did not undergo any additional freeze-thaw cycles.

### Proteomic analysis of urine samples

Pre-surgery urine samples were randomly selected from available AKI case (*n* = 9) and control (*n* = 7) patients and used for proteomic analysis. Urine samples were clarified of cells, bacteria or dispersed membrane fragments using sequential centrifugation, concentrated using Amicon Ultra-4 spin filters (10,000 MWCO membranes) and buffer exchanged into 0.01 M Hepes, 0.5 mM EDTA, pH 7.5 prior to proteomic analysis as previously described [29–31] using an LTQ-Orbitrap ELITE mass spectrometer. For comparative proteomics Scaffold Batch (v4.3.4) (ProteomeSoftware, Portland, OR) was used for label-free measurements based on both normalized MS2 spectral counting methods (NSAF) [32, 33] and MS1-based intensity based absolute quantification (iBAQ) [34] methods following correction for the false discovery rate using the Peptide and Protein Prophet algorithms [35, 36] and annotated with human gene ontology information from the Gene Ontology Annotations Database (ftp.ebi.ac.uk) [37]. Urinary proteins were analyzed using Ingenuity Pathways Analysis (IPA) software (http://ingenuity.com) to determine the extent of regulated proteins were enriched into known canonical biologic pathways or protein-protein interaction networks.

### Urine enzyme linked immunosorbent assays

Enzyme linked immunosorbent assays (ELISAs) were used to confirm quantitative differences in urine proteins on 47 subjects. The proteins were selected using a set of quantitative and qualitative filters to examine the spectral counting data for frequency of observation (observed in at least 80% of AKI positive and/or 80% of AKI negative samples), fold-change (increased or decreased by 2-fold with AKI), statistical importance by *p*-value (< 0.05) and biological importance by IPA pathways analysis. ELISA assays were conducted according to manufacturer protocols for use of urine and use of dilutions to bring analyte within calibration curve. C3 (ab108823) and Factor B (ab137973) kits were from Abcam (Cambridge, MA). CD59 (027694) and Histidine-rich glycoprotein (025685) kits were from US Biological (Salem, MA). Angiotensinogen (27412A kit was from Takara/Clonetech (Mountain View, CA).

### Statistical methods

Continuous variables were compared using two sample *t*-test. Biomarkers were tested for the prediction of AKI_R_ and AKI_KDIGO_ using univariate and multivariate logistic regression. Goodness of fit was determined by calculating the Cox and Snell R squared, area under the receiver operating curve (AUROC), and percent of corrected classifications. To evaluate the added effect of each candidate biomarker on AKI discrimination, we constructed receiver operator characteristic (ROC) curves and calculated the c-statistic, tested using integrated discrimination improvement (IDI), and net reclassification improvement (NRI) using the method developed by Pickering and Endre [38]. Both IDI and NRI are newer techniques to evaluate the incremental improvement in prediction over a baseline prediction model [39, 40]. The NRI evaluates the appropriateness of reclassification between models before and after the candidate biomarker is added, tabulating the frequency of appropriate versus inappropriate reclassification. A significant *P* value indicates that significantly more cases are being reclassified appropriately than inappropriately [41]. In contrast, the IDI determines how much an individual’s predicted risk changes with the use of different models [41]. IDI and NRI were compared to K_R_ and KDIGO risk scores. Statistics (ANOVA and post-hoc *t*-test) on LCMS data used normalized spectral counts and Scaffold Q + S Batch (ProteomeSoftware.com) software. Statistics on ELISA data and clinical parameters was by SPSS (ver24.0; Cary, NC).

## Results

### Patient population

A total of 47 patients from the 755 patients screened met eligibility requirements, provided written informed consent, underwent cardiac surgery, and had a complete set of data. The characteristics of those 47 patients who comprised this study are shown in Table 1. Of those patients 15 developed AKI_R_ based on a SCr level increase of ≥0.5 mg/dL or a ≥ 50% relative increase, while 32 did not meet that definition of AKI_R_. The age, race, and gender of those two groups were similar. Patients with AKI_R_ demonstrated a significantly higher risk factor score and a significantly higher pre-operative SCr level. None of the AKI_R_ patients required renal replacement therapy. There were no differences in the underlying pre-operative complications, pre-operative use of medications that interrupt the renin/angiotensin system, type of surgery performed, post-operative blood pressure, or post-operative urine output. Twenty-one subjects developed AKI using the KDIGO definition of AKI. Only peak SCr was different between patients with and without AKI_KDIGO_ (data not shown).

**Table 1:** Characteristics of patients with and without AKI

	AKI (15)	No AKI (32)	P value
Age	68 ± 11	67 ± 11	0.72
Male:Female	9:7	21:14	0.61
White:Black	11:4	29:5	0.20
Risk Factor Score	7.1 ±1.5	5.9 ± 1.1	0.005
CHF	8 (53)	25 (78)	0.083
DM	8 (53)	18 (56)	0.85
COPD	4 (27)	13 (41)	0.44
CKD	12 (80)	17 (53)	0.12
HTN	15 (100)	25 (78)	0.068
CVA	2 (13)	3 (9.4)	0.71
PVD	1 (6.7)	4 (13)	0.50
Pre-Op Creatinine (mg/dl)	2.1 ± 0.72	1.4 ± 0.59	0.002
Peak Creatinine (mg/dl)	3.4 ± 1.14	1.7 ± 0.62	<0.001
ACEI/ARB	3	16	0.026
Pre-Op SBP (mm/Hg)	141 ± 28	140 ± 21	0.91
Pre-Op DBP (mm/Hg)	78 ± 18.2	76 ± 13	0.71
6 hr SBP (mm/Hg)	106 ± 16	112 ± 16	0.25
6 hr DBP (mm/Hg)	51 ± 10	58 ± 21	0.23
CABG:valve:both	4:4:6	5:18:9	0.24
Urine Output 1^st^ 6 hr (ml)	444 ± 352	518± 223	0.40
Urine Output 1^st^ 24 hr (ml)	1256 ± 598	1567 ± 821	0.21
AKI Stage 1/2/3	13/2/0		

*CHF* congestive heart failure; *DM* diabetes mellitus; *COPD* chronic obstructive pulmonary disease; *CKD* chronic kidney disease; *HTN* hypertension; *CVA* cerebrovascular accident; *PVD* peripheral vascular disease; *ACEI* angiotensin converting enzyme inhibitor; *ARB* angiotensin receptor blocker; *SBP* systolic blood pressure; *DBP* diastolic blood pressure; *CABG* coronary artery bypass graft

### Proteomic analysis

**Fig. 1:**
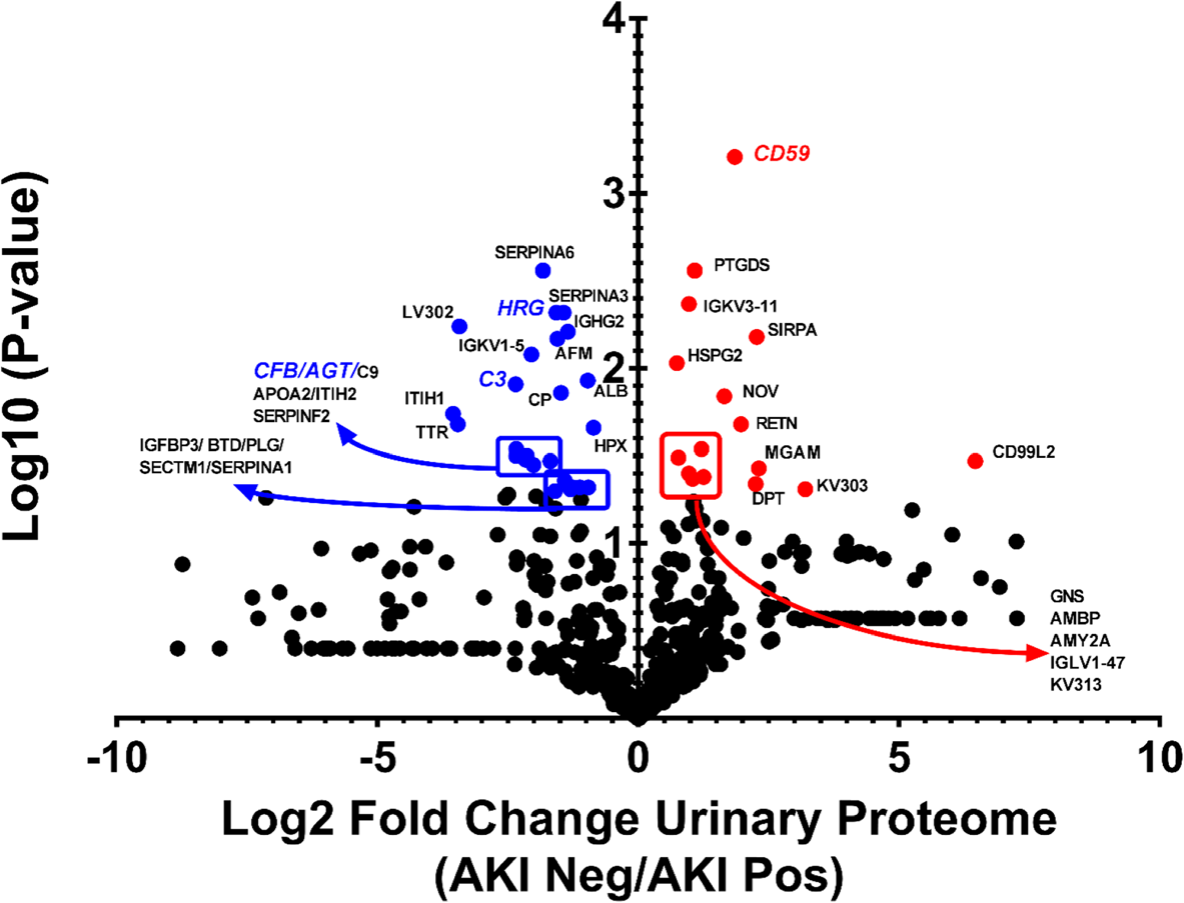
Volcano plot of pre-cardiac surgery urine protein differential abundance as assessed by significance (−Log10 of the *p*-value) versus Log2 of the fold change between the AKI Negative to AKI Positive urinary proteome. Data points are annotated by gene name for urinary proteins with a 2-fold change between groups and a p-value ≤0.05. Blue = higher abundance in AKI Positive urine samples. Red = higher abundance in AKI negative urine samples. Proteins selected for further ELISA tests have been bolded

Five hundred sixty-six identified proteins (Additional file 1) were curated to 160 proteins by eliminating all proteins that were not present in at least 80% either AKI_R_-positive or AKI_R_ -negative patients pre-surgery urine samples (Additional file 2). 56 proteins had a 2-fold or greater change; 38 were increased in AKI_R_ (+)and 18 proteins were increased in AKI_R_ (−) pre-surgical urine. When tested using a student’s *t*-test, 21 of 38 proteins increased in the AKI_R_ -positive group had a *p*-value < 0.05 (Additional file 3: Table S1A), and 8 of 18 proteins increased in the AKI_R_ -negative group had a *p*-value < 0.05 (Additional file 3: Table S1B). A volcano plot (Fig. 1) is presented and annotated with the final 29 protein data points by respective gene name. While kidney injury molecule 1 (KIM-1), neutrophil gelatinase-associated lipocalin (NGAL), liver-type fatty acid–binding protein (L-FABP), insulin-like growth factor–binding protein 7 (IGFBP7), and calprotectin (S100A8/S100A9) were observed within the original urine proteomic data set, none of those proteins differed statistically between AKI_R_ and non-AKI_R_ groups. Thus, those proteins were excluded from confirmatory studies. Based on the direction and magnitude of the Log2 fold change values for differential urinary abundance and the IPA analyses (Additional file 3: Table S2), angiotensinogen (AGT), complement factor C3 (C3), complement factor B (CFB), CD59 glycoprotein (CD59), and histidine rich glycoprotein (HRG) were selected for confirmatory ELISA studies.

### Risk prediction of urine biomarkers in AKI

**Fig. 2:**
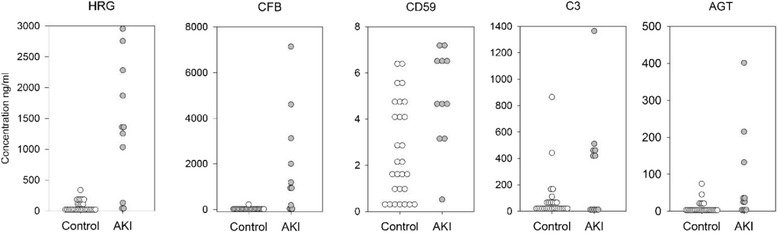
Box and whisker plots for pre-operative urine protein concentrations. Concentration units adjusted for urine dilution for CFB (ng/mL); CD59 (ng/mL); C3 (ng/mL); AGT (ng/mL); and HRG (ng/mL)

The urinary concentration of each of the 5 proteins was determined in all patients using ELISA assays (Fig. 2). The concentration of two proteins, HRG and CFB, was significantly higher in the AKI_R_ -positive group, compared to the AKI_R_ -negative group. Based on calibration curve analysis the performance of ELISA assays for HRG and CFB varied little between assay dates (HRG mean R^2^ 0.98 with a 0.2% CV; CFB mean R^2^ 0.99 with a 0.07% CV). Results of the univariate logistic regression analysis are shown in Table 2. HRG and CFB resulted in the best predictions of AKI_R_ with little difference in Cox and Snell R^2^ and no difference in the percent of correct classifications. Although statistically significant, both risk factor score and preoperative SCr had lower r^2^ values and between 8 and 13% lower correct classifications for patients with AKI_R_. Multivariate analysis demonstrated the utility of combining one or more of these factors in the logistic regression analysis. Combinations of risk factor score and either HRG or CFB resulted in the best predictions of AKI_R_ with 85.1 and 87.2% correct classifications, respectively (Table 2). The combination of all three factors together did not improve the overall fit. To evaluate the improvement of risk prediction with the addition of biomarkers to the established clinical model, we determined the NRI and the IDI indices. The NRI determines the appropriateness of reclassification of AKI risk between models before and after addition HRG or CFB. The IDI index determines the change in direction and amount of an individual’s predicted risk with the addition of HRG or CFB to the established model. Based on the IDI index, both HRG and CFB provided improved risk prediction over the pre-operative clinical model alone (Table 3). The IDI values show the magnitude of the improvement in prediction of AKI_R_ /no AKI_R_, was positive for both HRG (IDI = 0.34 CI 0.067 to 0.46) and Factor B (IDI = 0.35 CI 0.12 to 0.64) with the larger contribution to the total score due to the increased prediction of AKI_R_.

**Table 2:** Areas under the Receiver-Operating Characteristic Curve for Acute Kidney Injury for Univariate and Multivariate Predictor

Area Under the ROC curve (95% CI)	AKI	AKI_KDIGO_	P	P_KDIGO_
Univariate				
HRG	0.79 (0.65, 0.94)	0.79 (0.67, 0.92)	0.001	0.001
Preoperative Serum Creatinine	0.79 (0.66, 0.93)	0.63 (0.46, 0.79)	0.001	0.14
Factor B	0.75 (0.57, 0.93)	0.75 (0.60, 0.90)	0.007	0.004
Risk Factor Score	0.73 (0.56, 0.90)	0.62 (0.46, 0.79)	0.012	0.16
CD59	0.68 (0.50, 0.86)	0.67 (0.51, 0.84)	0.050	0.042
Angiotensinogen	0.65 (0.46, 0.84)	0.65 (0.48, 0.81)	0.11	0.091
C3	0.51 (0.3, 0.73)	0.59 (0.42, 0.77)	0.91	0.29
Multivariate				
HRG + Preoperative Serum Creatinine	0.82 (0.68, 0.96)	0.79 (0.66, 0.92)	<0.001	0.001
Factor B + Preoperative Serum Creatinine	0.84 (0.70, 0.98)	0.74 (0.59, 0.89)	<0.001	0.005
HRG + Factor B	0.79 (0.64, 0.94)	0.79 (0.66, 0.92)	0.002	0.001
HRG + Risk Factor Score	0.90 (0.79, 1.0)	0.79 (0.66, 0.92)	<0.001	0.001

*AKI*_*R*_, Restrictive AKI definition, *%Correct*_*R*_, percent of patients correctly classified using restrictive classification

*AKI*_*KDIGO*_, KDIGO AKI definition, *%Correct*_*KDIGO*_, percent of patients correctly classified using KDIGO classification

*P*_*R*_ *, p-value using restrictive AKI definition, P*_*KDIGO*_ *, p-value using KDIGO AKI definition*

**Table 3:** Net reclassification index* (NRI) and Integrated Discrimination Improvement (IDI) for the addition of HRG and Factor B when compared to Risk Score using the restrictive definition of AKI

	HRG	Factor B
Two-category NRI		
NRI events	33 (-13 to 69)	33 (-8.8 to 67)
NRI nonevents	6.3 (-2.5 to 24)	9.4 (0 to 35)
NRI	40 (-5.7 to 75)	43 (5.3 to 80)
IDI and summary statistics		
IDI events	0.23 (0.067 to 0.46)	0.24 (0.077 to 0.47)
IDI nonevents	0.11 (0.029 to 0.23)	0.11 (0.041 to 0.23)
IDI	0.34 (0.093 to 0.64)	0.35 (0.12 to 0.64)
IS ref	0.38 (0.21 to 0.54)	0.38 (0.22 to 0.54)
IS new	0.63 (0.39 to 0.82)	0.64 (0.43 to 0.81)
IP ref	0.23 (0.13 to 0.41)	0.23 (0.13 to 0.39)
IP new	0.15 (0.073 to 0.30)	0.15 (0.071 to 0.29)

Values in parenthesis are the 95% confidence intervals; NRI and IDI were calculated for the events, non-events, and total; *IS*, integrated sensitivity; *IP*, integrated 1-specificity *[39]

The ability of the five urinary proteins, pre-operative SCr, and risk factor score to predict postoperative AKI was determined for the 21 patients with the less restrictive KDIGO definition. Table 2 shows the results of the univariate logistic regression analysis. HRG and CFB were, again, the best predictors of AKI_KDIGO_ with an area under the ROC curve similar to that for AKI_R_. Risk factor score and preoperative SCr were no longer significantly predictive for AKI_KDIGO_. Multivariate analysis failed to find any utility of combining risk factor score or preoperative SCr with either HRG or CFB, nor did combining urine HRG and CFB improve prediction of either, alone, for AKI_KDIGO_ (Table 2). Percent correct classification was lower in the AKI_KDIGO_ group, compared to AKI_R_, for all variables, alone or in combination (Table 2).

## Discussion

Patients who develop AKI after cardiac surgery exhibit prolonged hospital stay, increased short-term and long-term mortality, and an increased risk of CKD [9]. As no effective treatment for established AKI exists, current clinical management focuses on risk factor assessment and prophylaxis. Sensitive and specific prediction of the risk of developing AKI is critical to identifying patients in whom the risk of AKI outweighs the benefits of surgery or in whom aggressive preoperative risk-reduction management is appropriate. The combination of preoperative laboratory and clinical evaluation was shown to be the best predictor of AKI whether or not dialysis was required [3]. That evaluation was highly predictive of AKI requiring dialysis with an AUROC of 0.93, however, the AUROC for AKI not requiring dialysis was only 0.75. As even milder forms of AKI are associated with worse short-term and long-term outcomes, improved risk assessment for AKI not requiring dialysis is needed. The current study tested the hypothesis that preoperative prognostic urinary biomarkers of post-surgical AKI_R_ could be identified using a proteomic approach and would be valuable adjunctive biomarkers for risk assessment. Six recognized diagnostic markers of AKI_R_ were detected, of which five (KIM1, NGAL, L-FABP, IGFBP7 and S100A8/S100A9) were not significantly different in patients developing AKI and one (AGT) was significantly different. AGT and four additional proteins (C3, CFB, HRG, IGFBP3), selected using rank ordering of abundance differences and pathways analysis, were studied by ELISA on the entire sample set. Two proteins in preoperative urine samples, HRG (AUROC 0.79) and CFB (AUROC 0.75), performed as well as the risk factor score (AUROC 0.73) and preoperative SCr (AUROC 0.79).

A multivariate model for the prediction of AKI_R_ performed better than any single factor measured. The addition of HRG or CFB to the risk factor score significantly improved the AUROC to 0.90 and 0.89, respectively. Differences in preoperative SCr observed between patients with and without AKI were corrected for in the model building process and did not explain the contribution of the newly discovered biomarkers to the prediction of AKI_R_. Despite the sensitivity of the CFB ELISA being at or near the measured concentration of nearly one-half of the samples analyzed, CFB still performed well in the multivariate model. CFB should not be ruled out as an important predictor of AKI_R_ and the development of a more sensitive assay could benefit this biomarker. The contribution of HRG and CFB to the improvement in the prediction of AKI_R_ (event) or no AKI_R_ (non-event) was evaluated using logistic regression and two statistical tests, NRI and IDI, developed specifically for the evaluation of potential biomarkers. NRI calculates the contribution using the binary values of 0 and 1 based on group membership (event, non-event) and IDI calculates the contribution based on probability 0.0 to 1.0 of the event occurring [38]. NRI and IDI were applied when factors were identified using logistic regression and were used to identify where prediction was improved (event, non-event, both) and as such are additive to the information displayed in the c-statistic. HRG and CFB influenced both the prediction of event (IDI only) and non-event (IDI only). The combined prediction (event + non-event) was also significant for CFB using both NRI and IDI. Use of HRG in the prediction of AKI_R_ resulted in an improvement in sensitivity over risk factor score from 0.38 to 0.63, a 25% improvement. There was improvement in 1-specificity from 0.23 to 0.15, an 8% improvement. Use of CFB in the prediction of AKI_R_ resulted in an improvement in sensitivity over risk factor score from 0.38 to 0.64, a 26% improvement, and an improvement in 1-specificity from 0.23 to 0.15, an 8% improvement.

The primary definition of AKI (AKI_R_) in the current study was more restrictive that those proposed using RIFLE, AKIN, or KDIGO criteria. We initially used a more restrictive definition, to reduce over diagnosis of AKI due to changes in fluid balance after cardiac surgery potentially leading to misleading changes in SCr or urine output [28]. Based on the improvement in predictive capability with CFB and HRG for AKI_R_, we re-analyzed predictive capability using the KDIGO criteria for AKI. That analysis showed that HRG and CFB continued to significantly predict development of AKI, although the prediction was less sensitive than for AKI_R_. Clinical risk factor score and preoperative SCr failed to predict AKI_KDIGO_. It is not possible to determine whether there was increased misdiagnosis of AKI using KDIGO criteria or if the predictors of AKI are less reliable for very mild cases.

CFB is a C3-convertase involved in alternative complement pathway activation and amplification [42, 43]. Genetic deletion of CFB or administration of anti-CFB monoclonal antibodies significantly impaired development of AKI in mice subjected to ischemia/reperfusion injury [44–46]. That reduction in CFB also significantly reduced the deposition of C3b on tubular epithelial cells and accumulation of neutrophils in the renal interstitium. Renal tubular cells showed increased CFB production in mice subjected to cecal ligation and puncture model of microbial sepsis and in cultured human proximal tubular cells stimulated with toll-like receptor agonists [46, 47]. Thus, increased CFB in the urine of patients undergoing cardiac surgery may identify those patients with underlying tubular cells CFB production that predisposes to complement-mediated tubular cell injury during surgery.

HRG is an abundant plasma glycoprotein with a multidomain structure that allows the molecule to interact with many ligands, including the complement components C1q, factor H, C8, C4, and C3 [48]. In addition to binding to a number of complement components, HRG was reported to inhibit complement factor D-mediated cleavage of CFB [49]. Although no association of HRG with AKI has been reported previously, the multiple protein-protein interactions with HRG regulates formation of immune complexes, removal of apoptotic cells, microbial invasion, cell adhesion, angiogenesis, coagulation, and progression of tumor growth [48].

The strengths of our study included a clear and restrictive definition of AKI using pre-operative and multiple post-operative serum and urine data to define the patient populations studied. A second strength of this study was utilization of high-sensitivity, high-mass accuracy proteomic methods to address the novel hypothesis that the pre-operative urine proteome was associated with post-cardiac surgery AKI. Importantly, confirmation of two candidate risk biomarkers using ELISA, an orthogonal method, was performed on all subjects. Those candidate biomarkers significantly enhanced the value of the clinical risk score for milder forms of AKI prediction. The clinical benefits of enhanced AKI prediction include: (1) improved application of prophylactic measures to a high risk population, (2) improved clinical assessment of the risk-to-benefit ratio of surgery, and (3) better patient cohort design for studies investigating AKI management and treatment.

Our study has some limitations. First, our data are specific to patients at higher risk for AKI who underwent cardiac surgery, and may not generalize as well to other patient populations. Additionally, using the risk factor score to focus our study on patients at higher risk skewed the scoring range in our population. This could confound the comparison of the risk factor score with urinary biomarkers. However, the AUROC for the risk factor score in our study (0.73) was similar to that previously reported in similar group of patients developing AKI not requiring dialysis (0.75) [3]. As patients in our study had AKI of mild severity, our urinary biomarkers may not improve risk prediction in patients with AKI requiring dialysis. Second, we did not study other biomarkers, except SCr, that have been associated with AKI following cardiac surgery. A previous study showed that pre-surgical serum cystatin C levels had a stronger and a more linear association with AKI risk than pre-surgical SCr [7]. A reduced urine uromodulin to SCr ratio was reported to be associated with an increased risk of AKI after cardiac surgery upon univariate, but not multivariate, analysis [50]. In neither report was the risk assessment determined by adding cystatin C or uromodulin values to the standard clinical risk assessment. Third, we did not have a validation set for our study, so confirmation will require further investigation. A larger cohort of unselected patients should generate a more powerful evaluation of whether HRG and CFB can improve risk discrimination for AKI.

## Conclusions

Pre-operative urine measurement of CFB or HRG significantly enhanced the current post-surgery AKI risk stratification. The ability of these biomarkers to predict risk for dialysis-requiring AKI or death could not be reliably assessed in our study due to a small number of patients with either outcome. If validated in future studies, our results suggest that urine concentration of HRG or CFB, alone or in addition to the past or current risk assessment tools [19, 27], will significantly enhance prediction of milder forms of AKI after cardiac surgery. In addition to providing improved clinical decision making, that enhanced prediction will assist patient selection in future studies of AKI management and treatment.

## Additional files

Additional file 1: Proteomic Data. Lists of all proteins identified with high confidence in pre-operative urine samples collected from AKI positive and AKI negative patients. Data are presented based on NSAF (normalized spectral abundance factor) estimations of relative abundance. Data include protein name, gene name, accession number, UniprotKB Entry Name, per patient NSAF values, group average NSAF, standard deviation of the average, and count based on fraction observed (n, %) as well as prevalence. Missing values were replaced by minimum observed NSAF value divided by the square root of 2. (PDF 505 kb)

Additional file 2: Proteomic Methods. Detailed methods and materials for urine sample handling, acquisition and analysis of LCMS data sets. (DOCX 26 kb)

Additional file 3: **Table S1A.** Proteins whose urinary abundance was decreased in patients with post-cardiac surgery AKI. Data are presented as gene symbols with associated relative abundance values (NSAF), *t*-test *p*-values, log2 fold changes, differences of relative abundance values and standard errors of the differences. **Table S1B.** Proteins whose urinary abundance was increased in patients with post-cardiac surgery AKI. Data are presented as gene names with associated relative abundance values (NSAF), *t*-test *p*-values, log2 fold changes, differences of relative abundance values and standard errors of the differences. Bolded and asterisk-marked entries represent targets selected for further confirmatory analysis by ELISA. **Table S2.** Ingenuity pathways analysis results for differentially abundance pre-surgical urine proteome listing statistically significant (*p*-value< 0.05) top networks and canonical pathways. Data are presented as network or pathway name with associated gene symbols and significance score for network (Z-score) and canonical pathway (*p*-value). Bolded and underlined entries represent targets selected for further confirmatory analysis by ELISA. (DOCX 17 kb)

## Abbreviations

AGT: Angiotensinogen
AKI: Acute kidney injury
AKIKDIGO: Acute kidney injury by KDIGO classification
AKIN: Acute Kidney Injury Network
AKIR: Acute kidney injury by restrictive classification
AUROC: Area under the receiver operating curve
C3: Complement factor C3
CABG: Coronary artery bypass grafting
CD59: CD59 glycoprotein
CFB: Complement factor B
CKD: Chronic kidney disease
ELISA: Enzyme linked immunosorbent assay
ESRD: End-stage renal disease
HRG: Histidine rich glycoprotein
iBAQ: Intensity based absolute quantification
IDI: Integrated discrimination improvement
IGFBP7: Insulin-like growth factor–binding protein 7
IPA: Ingenuity Pathways Analysis
KDIGO: Kidney Disease Improving Global Outcomes
KIM-1: Kidney injury molecule-1
L-FABP: Liver-type fatty acid–binding protein
NGAL: Neutrophil gelatinase-associated lipocalin NGAL
NRI: Net reclassification improvement
NSAF: Normalized spectral counting methods
RIFLE: Risk, Injury, Failure, Loss of kidney function, and End-stage kidney disease
ROC: Receiver operator characteristic
S100A8/S100A9: Calprotectin
SCr: Serum creatinine

## References

[1] Bastin AJ, Ostermann M, Slack AJ, Diller GP, Finney SJ, Evans TW (2013). Acute kidney injury after cardiac surgery according to risk/injury/failure/loss/end-stage, acute kidney injury network, and kidney disease: improving global outcomes classifications. J Crit Care.

[2] Dardashti A, Ederoth P, Algotsson L, Bronden B, Bjursten H (2014). Incidence, dynamics, and prognostic value of acute kidney injury for death after cardiac surgery. J Thorac Cardiovasc Surg.

[3] Kiers HD, van den Boogaard M, Schoenmakers MC, van der Hoeven JG, van Swieten HA, Heemskerk S, Pickkers P (2013). Comparison and clinical suitability of eight prediction models for cardiac surgery-related acute kidney injury. Nephrol Dial Transplant.

[4] Koyner JL, Garg AX, Coca SG, Sint K, Thiessen-Philbrook H, Patel UD, Shlipak MG, Parikh CR, Consortium T-A (2012). Biomarkers predict progression of acute kidney injury after cardiac surgery. J Am Soc Nephrol.

[5] Kuitunen A, Vento A, Suojaranta-Ylinen R, Pettila V (2006). Acute renal failure after cardiac surgery: evaluation of the RIFLE classification. Ann Thorac Surg.

[6] Robert AM, Kramer RS, Dacey LJ, Charlesworth DC, Leavitt BJ, Helm RE, Hernandez F, Sardella GL, Frumiento C, Likosky DS (2010). Cardiac surgery-associated acute kidney injury: a comparison of two consensus criteria. Ann Thorac Surg.

[7] Shlipak MG, Coca SG, Wang Z, Devarajan P, Koyner JL, Patel UD, Thiessen-Philbrook H, Garg AX, Parikh CR, Consortium T-A (2011). Presurgical serum cystatin C and risk of acute kidney injury after cardiac surgery. Am J Kidney Dis.

[8] Falvo A, Horst HM, Rubinfeld I, Blyden D, Brandt MM, Jordan J, Faber MD, Silverman N (2008). Acute renal failure in cardiothoracic surgery patients: what is the best definition of this common and potent predictor of increased morbidity and mortality. Am J Surg.

[9] Hobson CE, Yavas S, Segal MS, Schold JD, Tribble CG, Layon AJ, Bihorac A (2009). Acute kidney injury is associated with increased long-term mortality after cardiothoracic surgery. Circulation.

[10] Loef BG, Epema AH, Smilde TD, Henning RH, Ebels T, Navis G, Stegeman CA (2005). Immediate postoperative renal function deterioration in cardiac surgical patients predicts in-hospital mortality and long-term survival. J Am Soc Nephrol.

[11] Ryden L, Sartipy U, Evans M, Holzmann MJ (2014). Acute kidney injury after coronary artery bypass grafting and long-term risk of end-stage renal disease. Circulation.

[12] Elmistekawy E, McDonald B, Hudson C, Ruel M, Mesana T, Chan V, Boodhwani M (2014). Clinical impact of mild acute kidney injury after cardiac surgery. Ann Thorac Surg.

[13] Ishani A, Nelson D, Clothier B, Schult T, Nugent S, Greer N, Slinin Y, Ensrud KE (2011). The magnitude of acute serum creatinine increase after cardiac surgery and the risk of chronic kidney disease, progression of kidney disease, and death. Arch Intern Med.

[14] Lassnigg A, Schmidlin D, Mouhieddine M, Bachmann LM, Druml W, Bauer P, Hiesmayr M (2004). Minimal changes of serum creatinine predict prognosis in patients after cardiothoracic surgery: a prospective cohort study. J Am Soc Nephrol.

[15] Zakeri R, Freemantle N, Barnett V, Lipkin GW, Bonser RS, Graham TR, Rooney SJ, Wilson IC, Cramb R, Keogh BE (2005). Relation between mild renal dysfunction and outcomes after coronary artery bypass grafting. Circulation.

[16] Moran SM, Myers BD (1985). Pathophysiology of protracted acute renal failure in man. J Clin Invest.

[17] Bonventre JV, Weinberg JM (2003). Recent advances in the pathophysiology of ischemic acute renal failure. J Am Soc Nephrol.

[18] Molitoris BA (2003). Transitioning to therapy in ischemic acute renal failure. J Am Soc Nephrol.

[19] Thakar CV, Arrigain S, Worley S, Yared JP, Paganini EP (2005). A clinical score to predict acute renal failure after cardiac surgery. J Am Soc Nephrol.

[20] Englberger L, Suri RM, Li Z, Dearani JA, Park SJ, Sundt TM, Schaff HV (2010). Validation of clinical scores predicting severe acute kidney injury after cardiac surgery. Am J Kidney Dis.

[21] Wong B, St Onge J, Korkola S, Prasad B (2015). Validating a scoring tool to predict acute kidney injury (AKI) following cardiac surgery. Can J Kidney Health Dis.

[22] Parikh CR, Coca SG, Thiessen-Philbrook H, Shlipak MG, Koyner JL, Wang Z, Edelstein CL, Devarajan P, Patel UD, Zappitelli M (2011). Postoperative biomarkers predict acute kidney injury and poor outcomes after adult cardiac surgery. J Am Soc Nephrol.

[23] Parikh CR, Thiessen-Philbrook H, Garg AX, Kadiyala D, Shlipak MG, Koyner JL, Edelstein CL, Devarajan P, Patel UD, Zappitelli M (2013). Performance of kidney injury molecule-1 and liver fatty acid-binding protein and combined biomarkers of AKI after cardiac surgery. Clin J Am Soc Nephrol.

[24] Koyner JL, Vaidya VS, Bennett MR, Ma Q, Worcester E, Akhter SA, Raman J, Jeevanandam V, O'Connor MF, Devarajan P (2010). Urinary biomarkers in the clinical prognosis and early detection of acute kidney injury. Clin J Am Soc Nephrol.

[25] Bennett M, Dent CL, Ma Q, Dastrala S, Grenier F, Workman R, Syed H, Ali S, Barasch J, Devarajan P (2008). Urine NGAL predicts severity of acute kidney injury after cardiac surgery: a prospective study. Clin J Am Soc Nephrol.

[26] Arthur JM, Hill EG, Alge JL, Lewis EC, Neely BA, Janech MG, Tumlin JA, Chawla LS, Shaw AD, Investigators SA (2014). Evaluation of 32 urine biomarkers to predict the progression of acute kidney injury after cardiac surgery. Kidney Int.

[27] Englberger L, Suri RM, Li Z, Casey ET, Daly RC, Dearani JA, Schaff HV (2011). Clinical accuracy of RIFLE and acute kidney injury network (AKIN) criteria for acute kidney injury in patients undergoing cardiac surgery. Crit Care.

[28] Vives M, Wijeysundera D, Marczin N, Monedero P, Rao V (2014). Cardiac surgery-associated acute kidney injury. Interact Cardiovasc Thorac Surg.

[29] Wisniewski JR, Zougman A, Nagaraj N, Mann M (2009). Universal sample preparation method for proteome analysis. Nat Methods.

[30] Caster DJ, Korte EA, Merchant ML, Klein JB, Wilkey DW, Rovin BH, Birmingham DJ, Harley JB, Cobb BL, Namjou B (2015). Autoantibodies targeting glomerular annexin A2 identify patients with proliferative lupus nephritis. Proteomics Clin Appl.

[31] Hobeika L, Barati MT, Caster DJ, McLeish KR, Merchant ML (2017). Characterization of glomerular extracellular matrix by proteomic analysis of laser-captured microdissected glomeruli. Kidney Int.

[32] Zhang Y, Wen Z, Washburn MP, Florens L (2010). Refinements to label free proteome quantitation: how to deal with peptides shared by multiple proteins. Anal Chem.

[33] Zybailov B, Mosley AL, Sardiu ME, Coleman MK, Florens L, Washburn MP (2006). Statistical analysis of membrane proteome expression changes in Saccharomyces cerevisiae. J Proteome Res.

[34] Mann K, Edsinger E (2014). The Lottia gigantea shell matrix proteome: re-analysis including MaxQuant iBAQ quantitation and phosphoproteome analysis. Proteome Sci.

[35] Keller A, Nesvizhskii AI, Kolker E, Aebersold R (2002). Empirical statistical model to estimate the accuracy of peptide identifications made by MS/MS and database search. Anal Chem.

[36] von Haller PD, Yi E, Donohoe S, Vaughn K, Keller A, Nesvizhskii AI, Eng J, Li XJ, Goodlett DR, Aebersold R (2003). The application of new software tools to quantitative protein profiling via isotope-coded affinity tag (ICAT) and tandem mass spectrometry: II. Evaluation of tandem mass spectrometry methodologies for large-scale protein analysis, and the application of statistical tools for data analysis and interpretation. Mol Cell Proteomics.

[37] Ashburner M, Ball CA, Blake JA, Botstein D, Butler H, Cherry JM, Davis AP, Dolinski K, Dwight SS, Eppig JT (2000). Gene ontology: tool for the unification of biology. The gene ontology consortium. Nat Genet.

[38] Pickering JW, Endre ZH (2012). New metrics for assessing diagnostic potential of candidate biomarkers. Clin J Am Soc Nephrol.

[39] Pencina MJ, D'Agostino RB, D'Agostino RB, Vasan RS (2008). Evaluating the added predictive ability of a new marker: from area under the ROC curve to reclassification and beyond. Stat Med.

[40] Cook NR (2008). Statistical evaluation of prognostic versus diagnostic models: beyond the ROC curve. Clin Chem.

[41] Lloyd-Jones DM (2010). Cardiovascular risk prediction: basic concepts, current status, and future directions. Circulation.

[42] Mathern DR, Heeger PS (2015). Molecules great and small: the complement system. Clin J Am Soc Nephrol.

[43] Ricklin D, Reis ES, Lambris JD (2016). Complement in disease: a defence system turning offensive. Nat Rev Nephrol.

[44] Thurman JM, Ljubanovic D, Edelstein CL, Gilkeson GS, Holers VM (2003). Lack of a functional alternative complement pathway ameliorates ischemic acute renal failure in mice. J Immunol.

[45] Thurman JM, Lucia MS, Ljubanovic D, Holers VM (2005). Acute tubular necrosis is characterized by activation of the alternative pathway of complement. Kidney Int.

[46] Zou L, Feng Y, Li Y, Zhang M, Chen C, Cai J, Gong Y, Wang L, Thurman JM, Wu X (2013). Complement factor B is the downstream effector of TLRs and plays an important role in a mouse model of severe sepsis. J Immunol.

[47] Li D, Zou L, Feng Y, Xu G, Gong Y, Zhao G, Ouyang W, Thurman JM, Chao W (2016). Complement factor B production in renal tubular cells and its role in sodium transporter expression during Polymicrobial Sepsis. Crit Care Med.

[48] Poon IK, Patel KK, Davis DS, Parish CR, Hulett MD (2011). Histidine-rich glycoprotein: the Swiss Army knife of mammalian plasma. Blood.

[49] Chang NS, Leu RW, Rummage JA, Anderson JK, Mole JE (1992). Regulation of complement functional efficiency by histidine-rich glycoprotein. Blood.

[50] Garimella PS, Jaber BL, Tighiouart H, Liangos O, Bennett MR, Devarajan P, El-Achkar TM, Sarnak MJ (2017). Association of Preoperative Urinary Uromodulin with AKI after cardiac surgery. Clin J Am Soc Nephrol.

