# Supplementary material for: Biomarker enhanced risk prediction for development of AKI after cardiac surgery
Source: BMC Nephrol. 2018 May 2;19:102. doi: 10.1186/s12882-018-0902-9 (PMC5930948; doi:10.1186/s12882-018-0902-9)
Supplement: Supplementary file 2 — Proteomic Methods. Detailed methods and materials for urine sample handling, acquisition and analysis of LCMS data sets. (DOCX 26 kb) [file 12882_2018_902_MOESM2_ESM.docx]

Supporting Proteomic Methods for-

Biomarker Enhanced Risk Prediction for Development of AKI after Cardiac Surgery

### Proteomic Analysis of Urine Samples

### Pre-surgery urine samples from AKI case (n=9) and control (n=7) patients were used for proteomic analysis. Urine samples were spun first at 1,500xg for 5min at 4 C then upper liquid portion spun in fresh tubes at 17,000xg for 15min at 4 C to pellet any cells, bacteria or cell debris. Supernatants were transferred to ultracentrifugation tubes and spun at 200,000xg for 1hr at 4C to clarify the urine of colloidal material such as urinary extracellular microvesicles. Using Amicon Ultra-4 spin filters (10,000 MWCO membranes), urine samples were then concentrated 10x and then rinsed (three times) to buffer exchange into 0.01M Hepes, 0.5mM EDTA, pH 7.5. The concentrated urine protein is recovered from the Amicon filtration device by pipetting. A 0.02% acid-labile surfactant solution (ProteaseMAX, Promega, Madison, WI) dissolved in 0.01M Hepes, 0.5mM EDTA, pH 7.5 was used to recover any residual protein from the cellulosic membrane and combined with the recovered urine sample. The protein samples were assayed using a Bradford method and samples (100ug) were reduced with 0.1M DTT for 60°C for 30min prior to dilution into Filter-Aided Sample Preparation (FASP) buffer (8M urea, 0.1M Tris-HCl pH8.5) and transferred to a Microcon-10 (EMD Millipore, Billerica, MA) 10kDa centrifugal for trypsinization by the FASP method.(1)

### Tryptic digests were trap cleaned using C18 PROTOTM Ultra MicroSpin columns (Nest Group, Inc, Southborough, MA) then lyophilized and redissolved into 2% acetonitrile/0.1% formic acid prior to off-line strong cation exchange (SCX) fractionation into seven fractions as described previously. (2) Tryptic peptides (0.5ug) were separated using an EASY n-LC (Thermo) UHPLC system and a 360µm OD x 100µm ID fused silica tip packed with 10cm of Jupiter 5µm C18 300Å material (Phenomenex, Torrance, CA). Following injection of the sample onto the column, separation was accomplished with a 75min linear gradient from 2% acetonitrile to 40% acetonitrile in 0.1% formic acid.

### The eluate was introduced into the LTQ-Orbitrap ELITE mass spectrometer using a Nanospray Flex source (ThermoElectron, Waltham, MA) and data was collected with an Nth Order Double Play with ETD Decision Tree method created in Xcalibur v2.2.(3) Scan event one of the method obtained an FTMS MS1 scan (normal mass range; 60,000 resolution, full scan type, positive polarity, profile data type) for the range 300-2000m/z. Scan event two through ten obtained ITMS MS2 scans (normal mass range, rapid scan rate, centroid data type) on peaks that had a minimum signal threshold of 20,000 counts from scan event one. A decision tree was used to determine whether CID or ETD activation was used. An ETD scan was triggered if any of the following held: an ion had charge state 3 and m/z less than 650, an ion had charge state 4 and m/z less than 900, an ion had charge state 5 and m/z less than 950, or an ion had charge state greater than 5; a CID scan was triggered in all other cases. The lock mass option was enabled (0% lock mass abundance) using the 371.10124m/z polysiloxane peak as an internal calibrant.

### Data dependent spectra search was conducted using SageN Sorcerer 2 Sequest and X!Tandem algorithm sand the UniprotKB Homo sapiens reference proteome canonical and isoform sequences (11/07/2014 version; 88808 entries). Search parameters included: variable methionine oxidation, fixed cysteine carbamidomethylation, up to 2 missed tryptic cleavages, 10ppm precursor error for MS1 Orbitrap FTMS data, 1 Da error for MS2 data sets. For comparative proteomics Scaffold Batch (v4.3.4) (ProteomeSoftware, Portland, OR) was used for label-free measurements based on both normalized MS2 spectral counting methods (NSAF)(4, 5) and MS1-based intensity based absolute quantification (iBAQ)(6) methods following correction for the false discovery rate using the Peptide and Protein Prophet algorithms (7, 8) and annotated with human gene ontology information from the Gene Ontology Annotations Database (ftp.ebi.ac.uk).(9) Urinary proteins were analyzed using Ingenuity Pathways Analysis (IPA) software (<http://ingenuity.com>) to determine emergent trends in protein urinary abundance patterns.

### Missing data

### Censored or missing values were then replaced by a minimal global protein abundance value divided by the square root of two (Minimal observed label-free signal÷√2) or 3.6E-05.(10)

**Data sharing**

Data files for acquired LCMS data (.RAW), for peak lists (.mgf), and compressed search results (.mzIdentML) files will be deposited in MassIVE (http://massive.ucsd.edu/) data repository with the Center for Computational Mass Spectrometry at the University of California, San Diego and shared with the ProteomeXchange (www.proteomexchange.org).

1. Wisniewski JR, Zougman A, Nagaraj N, Mann M: Universal sample preparation method for proteome analysis. *Nat Methods,* 6**:** 359-362, 2009

2. Uriarte SM, Rane MJ, Merchant ML, Jin S, Lentsch AB, Ward RA, McLeish KR: Inhibition of neutrophil exocytosis ameliorates acute lung injury in rats. *Shock,* 39**:** 286-292, 2013

3. Swaney DL, McAlister GC, Coon JJ: Decision tree-driven tandem mass spectrometry for shotgun proteomics. *Nat Methods,* 5**:** 959-964, 2008

4. Zhang Y, Wen Z, Washburn MP, Florens L: Refinements to label free proteome quantitation: how to deal with peptides shared by multiple proteins. *Anal Chem,* 82**:** 2272-2281, 2010

5. Zybailov B, Mosley AL, Sardiu ME, Coleman MK, Florens L, Washburn MP: Statistical analysis of membrane proteome expression changes in Saccharomyces cerevisiae. *J Proteome Res,* 5**:** 2339-2347, 2006

6. Mann K, Edsinger E: The Lottia gigantea shell matrix proteome: re-analysis including MaxQuant iBAQ quantitation and phosphoproteome analysis. *Proteome Sci,* 12**:** 28, 2014

7. Keller A, Nesvizhskii AI, Kolker E, Aebersold R: Empirical statistical model to estimate the accuracy of peptide identifications made by MS/MS and database search. *Anal Chem,* 74**:** 5383-5392, 2002

8. von Haller PD, Yi E, Donohoe S, Vaughn K, Keller A, Nesvizhskii AI, Eng J, Li XJ, Goodlett DR, Aebersold R, Watts JD: The application of new software tools to quantitative protein profiling via isotope-coded affinity tag (ICAT) and tandem mass spectrometry: II. Evaluation of tandem mass spectrometry methodologies for large-scale protein analysis, and the application of statistical tools for data analysis and interpretation. *Mol Cell Proteomics,* 2**:** 428-442, 2003

9. Ashburner M, Ball CA, Blake JA, Botstein D, Butler H, Cherry JM, Davis AP, Dolinski K, Dwight SS, Eppig JT, Harris MA, Hill DP, Issel-Tarver L, Kasarskis A, Lewis S, Matese JC, Richardson JE, Ringwald M, Rubin GM, Sherlock G: Gene ontology: tool for the unification of biology. The Gene Ontology Consortium. *Nat Genet,* 25**:** 25-29, 2000

10. Succop PA, Clark S, Chen M, Galke W: Imputation of data values that are less than a detection limit. *J Occup Environ Hyg,* 1**:** 436-441, 2004
