# Supplementary material for: Biomarker enhanced risk prediction for development of AKI after cardiac surgery
Source: BMC Nephrol. 2018 May 2;19:102. doi: 10.1186/s12882-018-0902-9 (PMC5930948; doi:10.1186/s12882-018-0902-9)
Supplement: Supplementary file 3 — Table S1A. Proteins whose urinary abundance was decreased in patients with post-cardiac surgery AKI. Data are presented as gene symbols with associated relative abundance values (NSAF), t-test p-values, log2 fold changes, differences of relative abundance values and standard errors of the differences. Table S1B. Proteins whose urinary abundance was increased in patients with post-cardiac surgery AKI. Data are presented as gene names with associated relative abundance values (NSAF), t-test p-values, log2 fold changes, differences of relative abundance values and standard errors of the differences. Bolded and asterisk-marked entries represent targets selected for further confirmatory analysis by ELISA. Table S2. Ingenuity pathways analysis results for differentially abundance pre-surgical urine proteome listing statistically significant (p-value< 0.05) top networks and canonical pathways. Data are presented as network or pathway name with associated gene symbols and significance score for network (Z-score) and canonical pathway (p-value). Bolded and underlined entries represent targets selected for further confirmatory analysis by ELISA. (DOCX 17 kb) [file 12882_2018_902_MOESM3_ESM.docx]

| Supplemental Table 1A. Decreased urinary abundance associated with post-cardiac surgery AKI | | | | | | |
| --- | --- | --- | --- | --- | --- | --- |
| Protein ID (Gene Name) | P value (T-test) | AKI Neg. Pre  (mean NSAF)  (x10^-3^) | AKI Pos. Pre  (mean NSAF)  (x10^-3^) | LOG2 Fold Change  (AKI-Pos/AKI-Neg) | Difference  (x10^-2^) | SE of difference  (x10^-3^) |
| SIRPA | 0.0066 | 1.5 | 0.30 | -2.3 | -0.12 | 0.40 |
| RETN | 0.021 | 7.0 | 1.8 | -2.0 | -0.51 | 2.0 |
| **CD59*** | 0.00061 | 28 | 7.9 | -1.9 | -2.1 | 4.7 |
| NOV | 0.014 | 1.7 | 0.53 | -1.7 | -0.11 | 0.40 |
| AMY2A | 0.042 | 4.1 | 1.7 | -1.3 | -0.24 | 1.1 |
| GNS | 0.029 | 18. | 0.78 | -1.2 | -0.10 | 0.40 |
| PTGDS | 0.0028 | 54. | 26. | -1.1 | -2.9 | 7.9 |
| IGLV1-47 | 0.043 | 6.1 | 3.0 | -1.0 | -0.32 | 1.4 |
|  |  |  |  |  |  |  |
| Supplemental Table 1B. Increased urinary abundance associated with post-cardiac surgery AKI | | | | | | |
| Protein ID (Gene Name) | P value (T-test) | AKI Neg. Pre (mean NSAF)  (x10^-3^) | AKI Pos. Pre (mean NSAF)  (x10^-3^) | LOG2 Fold Change  (AKI-Pos/AKI-Neg) | Difference  (x10^-2^) | SE of difference  (x10^-3^) |
| TTR | 0.021 | 0.50 | 5.6 | 3.5 | 0.50 | 1.9 |
| LV302 | 0.0059 | 0.15 | 1.6 | 3.4 | 0.14 | 0.40 |
| **C3*** | 0.012 | 0.87 | 4.4 | 2.4 | 0.36 | 1.2 |
| SERPINF2 | 0.032 | 0.18 | 0.93 | 2.3 | 0.075 | 0.30 |
| APOA2 | 0.029 | 0.49 | 2.5 | 2.3 | 0.20 | 0.80 |
| C9 | 0.033 | 0.22 | 1.0 | 2.2 | 0.077 | 0.30 |
| ITIH2 | 0.032 | 0.13 | 0.59 | 2.1 | 0.045 | 0.20 |
| IGKV1-5 | 0.0083 | 0.37 | 1.5 | 2.1 | 0.12 | 0.40 |
| **AGT*** | 0.035 | 0.66 | 2.7 | 2.0 | 0.2 | 0.90 |
| SERPINA6 | 0.0028 | 1.1 | 4.0 | 1.8 | 0.29 | 0.80 |
| **CFB*** | 0.034 | 0.44 | 1.4 | 1.7 | 0.097 | 0.40 |
| BTD | 0.050 | 0.33 | 1.0 | 1.6 | 0.067 | 0.30 |
| **HRG*** | 0.0048 | 0.60 | 1.8 | 1.6 | 0.12 | 0.40 |
| AFM | 0.0067 | 0.76 | 2.2 | 1.6 | 0.15 | 0.5 |
| CP | 0.014 | 1.8 | 4.9 | 1.5 | 0.32 | 1.1 |
| SERPINA3 | 0.0048 | 3.7 | 9.9 | 1.4 | 0.62 | 1.9 |
| PLG | 0.044 | 1.0 | 2.7 | 1.4 | 0.17 | 0.80 |
| IGHG2 | 0.0062 | 7.6 | 19. | 1.4 | 1.2 | 3.7 |
| SERPINA1 | 0.049 | 9.2 | 23. | 1.3 | 1.3 | 6.2 |
| APOH | 0.048 | 1.8 | 4.3 | 1.3 | 0.25 | 1.1 |
| **IGFBP3*** | 0.048 | 0.38 | 0.82 | 1.1 | 0.044 | 0.20 |

Supplemental Table 2. Analysis of emergent properties using Ingenuity Pathways Analysis of the twenty-nine (29) regulated proteins by top networks and top canonical pathways. Bolded entries with an asterisk (*) were quantified by ELISA.

Top Networks Score No. Proteins

1. Cell-to-Cell Signaling and Interaction, Hematological 24 12

System Development and Function, Organismal Functions, Immune Cell Trafficking

(APOH, **CFB**, IGFBP3**,** IGHG2, NOV, PLG, RETN, SERPINA1, SERPINF2, SIRPA, TTR)

1. Cellular Movement, Hematological System Development and 16 9

Function, Immune Cell Trafficking

(**AGT**, APOA2, **C3,** C9**, CD59, HRG,** PLG, PTGDS, SERPINA3)

Canonical Pathways (p-value) No. Proteins

1. Acute Phase Response Signaling (1.44 E-22) 14/169 (8.3%)

(**AGT**, APOA2, APOH, **C3,** C9, **CFB**, CP, **HRG**, ITIH2, PLG, SERPINA1, SERPINF2, SERPING3, TTR)

1. LXR/RXR Activation (3.53 E-12) 8/121 (6.6%)

(**AGT**, APOA2, APOH, **C3,** C9, SERPINA1, SERPINF2, TTR)

1. FXR/RXR Activation (4.59 E-12) 8/125 (6.4%)

(**AGT**, APOA2, APOH, **C3,** C9, C4B, SERPINA1, SERPINF2, TTR)

1. Complement System (1.67 E-07) 4/36 (11.1%)

(**C3,** C9**,** **CD59, CFB**)
